# The effectiveness of the department of defense’s field manual 3-11 in detecting, deterring and degrading the breach of a combat base by a human-borne with bioagent (HBBA): perceptions of security personnel

**DOI:** 10.1186/s40779-015-0065-y

**Published:** 2015-12-22

**Authors:** George Edafese Alakpa, John W. Collins

**Affiliations:** Department of Professional Security Studies, New Jersey City University, Jersey City, NJ USA

**Keywords:** DoD FM 3-11, Bioterrorism, Breach of Combat ECP, HBBA terrorist, Security personnel, Perception

## Abstract

**Background:**

The department of defense’s FM 3-11 is among the military’s field manuals for preparing for, reacting to and recovering from chemical, biological, radiological and nuclear attacks. Since post 9-11, U.S. military service members have been deployed in the global war on terrorism. This study attempted to determine the effectiveness of the FM 3-11 in detecting, deterring or preventing a human-borne with bioagent (HBBA) terrorist breach at an entry control point (ECP).

**Method:**

This time-specific, cross-sectional study disseminated a validated survey tool with Cronbach’s α > 0.82 to respondents who have had antiterrorism training and combat ECP experience. The return rate was greater than 75.0 %; however, many of the respondents failed to meet the inclusion criteria. Consequently, only 26 questionnaires were included in the sample.

**Results:**

The results revealed that while over 60.0 % of the respondents either strongly agreed or agreed that biointelligence, the deployment of biodetectors and the use of biowarning systems could be effective in preventing an ECP breach by a terrorist with a bioagent, the use of protective equipment and immunization to decontaminate service members or other TTPs would never prevent a breach. A large percentage of respondents claimed that soldiers at the ECP lacked the devices or the knowledge to detect an HBBA at an ECP, and 72.0 % suggested modifying current ECP TTPs to include education, training and equipment for security personnel at military base ECPs.

**Conclusion:**

If obtained from appropriate sources and communicated to the personnel at the ECP in an effective or timely manner, the possible effectiveness of certain TTPs in the FM 3-11, specifically FM 3-11.86 (intelligence), might increase.

## Background

The September 11, 2001 attack on the United States and the anthrax incidents that same year are generally agreed to have changed the global and U.S. response to terrorism; as a result, the “nation’s bioterrorism response capability has become an imminent priority for policymakers, researchers, public health officials, academia and the private sector” ([1], p.1). Previously, in 1984, a religious cult called “Bagwan Shree Rajneesh” was reported to have contaminated a salad bar in the U.S. with Salmonella in the first case of bioterrorism (BT). Other cases include the 1996 *Shigella dysenteriae* Type 2 contamination of muffins and donuts in Dallas, Texas, and the 1997 and 1998 anthrax hoaxes reported in Washington, DC, and Los Angeles, respectively [2–4].

Other bioterrorist events, as reported by Tucker [5], include the 1970 “Weather Underground” revolutionary group attack on federal buildings; the 1972 college ecoterrorist group, “R.I.S.E”, which employed eight microbial pathogens, including typhoid fever, diphtheria, dysentery and meningitis; the 1980 attack by the “Red Army Fraction” group, a Marxist revolutionary ideological group; the deliberate contamination of salad bars with Salmonella bacterium 1984 by a “Rajneeshee Cult”; a in 1991 ricin threat made by the “Minnesota Patriots Council” for personal revenge; and, in 1998, the arrest of Larry Wayne Harris when he talked about obtaining and deploying anthrax to achieve a “white supremacist goal”. Dudley [6] reported that between 1990 and 2000, a total of 1368 cases of tularemia, one of the recognized diseases caused by a bioagent *Francisella tularensis* (a biological agent), were reported in the U.S.

In March 2002 in Texas, the 12th cutaneous anthrax case was detected and linked to mail in a Texas laboratory. In 2003, a total of nine ricin biothreats were reported [7], while on February 3, 2004, the Dirksen Senate Office Building in Washington, DC, was reported to have discovered ricin in the office of Senator Bill Frist. In April, 2013, letters that tested positive for ricin were reportedly sent to Senator Roger Wicker [8], and similar letters were also reportedly sent to the President of the United States, Barack Obama, and the then-mayor of New York City, Michael Bloomberg [9].

### Field manuals, FM 3-11

The U.S. military has compiled and published many sets of procedures as manuals for practically every operation and for those employed in the field. These manuals include, but are not limited to, the Joint Entry Control Point & Escalation of Force Procedures (JEEP) [10], the Joint Forward Operations Base (JFOB) [11], the Unit Antiterrorism Officer (UAO) Handbook [12] and the Field Manual (FM) 3-11 [13]. Of these manuals, the FM 3-11 series is the most specific to biological agent preparation and reactions; thus, it is a focus for critique in this study. This multi-service tactics, techniques and procedure (TTP) manual focus on chemical, biological, radiological and nuclear (CBRN) agents. There are approximately six different subclasses of this manual, numbered 3-11, 3-11.3; 3-11.4; 3-11.5; 3-11.86 and 3-11.9 [13–18]. In summary, FM 3-11 covers the military’s strategies and policy on CBRN; FM 3-11.3, updated in 2009, addresses steps for avoiding CBRN agents; FM 3-11.4 addresses protective wear and measures and was updated in 2009. FM 3-11.5 details decontamination procedures, FM 3-11.86 emphasizes the roles of biological surveillance, and FM 3.11-9 provides a detailed reference for the biological and chemical agents employed for CB warfare [13–18]. While these TTPs are very useful, they are not specific enough for easy application at the entry control point (ECP).

The U.S. military is one of the major resources the government employs for national security [19] and the global war on terrorism; consequently, service members (SMs) are exposed to the possibility of a bioagent attack. This exposure was anticipated during the invasion of Iraq in 1990 and 2003, when SMs were given mandatory antibiotics [20]. The exact number of military bases in foreign location is ever-changing because of the dynamics of the nation’s anti-terrorism strategy; however, the Department of Defense’s (DoD) 2010 report indicates that there were 662 facilities maintained by the U.S. military in 38 foreign countries, excluding those in Iraq and Afghanistan [21].

Totten [22] reported that returning U.S. military personnel, like international travelers, immigrants, or imported goods, are viewed even by intelligence agencies as sources for the introduction of contagious infectious diseases into the U.S. As multi-national forces engage in joint missions in regional and global attempts to address conflicts or humanitarian crises, questions are arising about the possible roles the military may play in disseminating diseases in the countries where they are deployed or in the acquisition of diseases from fellow soldiers from other nations during joint deployments [23, 24]. Aerssens’ 2011 study [23] reported the Belgian troops’ return from Congo with parasite infections.

Many studies have been conducted by the military in the U.S. and other countries to understand the role and impact of infectious disease dissemination among deployed personnel in terms of disease surveillance, [25–27], but at present, no study has addressed the breach of an ECP with the intent to cause bioterrorism (BT) against U.S. military personnel that would impact their health and mission.

On September 16, 2014, United States President Barack Obama announced to the nation his strategy and actions to combat the Ebola outbreak in some countries in the West African countries of Liberia, Sierra Leone and Guinea, with a promise to deploy U.S. military service members [28]. Immediately, questions from members of press reflected fears about the possibilities of these military men and women becoming infected with the virus and becoming “vectors” of transmission back to the USA.

While at this time, there is no documented evidence of an HBBA terrorist attack against a U.S. military combat, numerous cases of possible “potential vulnerabilities” have been reported by Hylton [29], and more have been reported by Alakpa [30]. The lack of such an attack does not rule out the future use of such an insidiously deadly form of terrorism to threaten the U.S. security and its forces stationed abroad. Should a terrorist group attempt this route of attack, what measures are in place to check or prevent a breach of an FOB’s ECP?

The purpose of this study was to determine whether the current DoD FM 3-11 field manual series, which is specifically designed to prepare for and respond to a biological agent attack, would be effective for detecting, deterring and degrading a terrorist with a BA at a military base ECP.

## Method

### Respondents

The target populations selected for this pretest were U.S. military personnel (primarily) and other individuals in the security profession (secondarily) with combat experience. A total of 110 questionnaires were distributed, including single copies given to the 13 points of contact (POCs) for willing respondents at eight military installations: a National Guard post, three police stations in Sussex County, NJ, the Veteran’s Administration (VA) Security Post at Castle Point, NY, two military/veteran coordinators at NJ Universities, and the U.S. Customs and Borders Office in Newark, NJ.

### Research design

This was a cross-sectional survey study with the administration of a validated, five-point Likert-scaled questionnaire with two constructs and Cronbach’s alpha reliabilities of 0.820 and 0.892 for Constructs 1 and 2, respectively [30]. The questionnaire was distributed to respondents in the selected organizations, and a single-blind approach was used to ensure that no direct contact occurred between the respondents and investigator. However, in some situations, there was direct contact between prospective respondents (those willing to participate) and the investigator, who later offered these respondents hard copies of the questionnaires, along with consent forms. Electronic copies were also sent to respondents who requested the questionnaire and consent forms in an electronic format.

### Data collection

Data from the target population was collected with the validated survey tool. Only those questionnaires that were fully completed or had no more than four missing items and in which the respondents demonstrated knowledge or training on antiterrorism ECP TTPs were accepted for analysis.

### Data analysis

Data collected were analyzed using the Statistical Product and Service Solution (SPSS) statistical software (Base Grad Pack shrink wrap version 21.0) for both descriptive and scale reliability—Cronbach’s alpha analysis.

### Delimitations of study

This research study focused primarily on military combat settings. The respondents were primarily military or involved in national security and had personal knowledge of and/or training in antiterrorism (AT) measures and TTPs. It did not reveal or expose details about classified military TTPs, analyze or review specifics of the military TTPs that focus on reaction and recovery from terrorist attacks on combat bases, or reveal exact names of combat bases and their coordinates or the specifics of their activities. The study was limited to the periods witnessed by one of the investigators while on tour of duty in Afghanistan as a force protection vulnerability assessor for the U.S. Army Central Command.

### Ethical statement/approval

The New Jersey City University IRB approved this research on 05/13/2014 as part of the corresponding author’s DSc dissertation. The respondents’ privacy was protected and no identifying personal information was collected.

## Results

Only questionnaires that were at least 95.0 % complete and respondents who demonstrated personal knowledge of or training in any anti-terrorism (AT) tactics, techniques and procedures (TTP) were included in the analyses. The return rate from those who were willing to participate and signed the consent form was 75.0 %. After eliminating those who did not fit the required criteria, 26 questionnaires that could be subjected to analysis remained.

### Limitations of results

During the dissemination of the survey tool (questionnaire) to the target population, many in the military or Custom and Border Control/Immigration Service agents showed reluctance to participate or permit their subordinates to participate. This reluctance ultimately affected the response rate, the number of respondents, and eventually the sample size of this pretest study.

This small sample size made it impossible for the researchers to make a broad generalization or inference from the findings of the study. However, it is important to emphasize that the results tend to show that the ECPs of combat FOBs are vulnerable to breach by a terrorist carrying biological agents. Additionally, these perceptions come from people who have generally been recently deployed and have ECP TTP experiences in a combat environment. Field experience is an important factor in these positions, as individuals who have not been deployed lack the necessary readiness. Over 92 % of the respondents in this study whose questionnaires were completed, returned and analyzed were combat veterans with a minimum of two tours of deployment and with ECP TTP personal experience.

### Descriptive data of respondents

More than eighty percent (80.8 %) of respondents whose questionnaires were selected and analyzed were in the military; 15.4 % were retired military, and 3.8 % were from Homeland Security. In terms of military service branch, 80.0 % were in the U.S. Army, 12.0 % were in the U.S. Air Force, 4.0 % were in the U.S. Navy, and 4.0 % were U.S. Marines. Of this sample, 69.2 % were enlisted, 26.9 % were officers, and 3.8 % were civilians.

### Combat experience of the respondents

More than ninety-two percent (92.3 %) of the respondents had been deployed to combat zones, with 75.0 % just returning from the Operation Enduring Freedom (OEF) campaign in Afghanistan; 16.7 % were in the Iraq war, and 8.3 % were involved in Operation New Dawn. All of the respondents claimed to have had personal knowledge of or training in anti-terrorism.

#### Respondents’ perceptions of the effectiveness of CBRN TTP

**Fig. 1:**
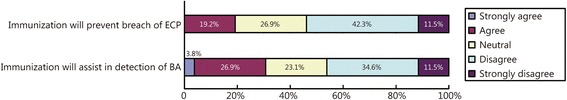
The respondents’ perceptions regarding the effectiveness of immunization protocols against a BA breach at an ECP

Slightly more than forty-six percent (46.1 %) of respondents strongly disagreed or disagreed with the notion that immunizing every resident of a combat post will assist in the detection of a terrorist with a BA at the ECP. Additionally, 53.8 % strongly disagreed or disagreed that immunization would prevent the breach of a combat ECP by a terrorist with a BA (Fig. 1).

#### Biosurveillance TTP as detailed in FM 3-11.86

**Fig. 2:**
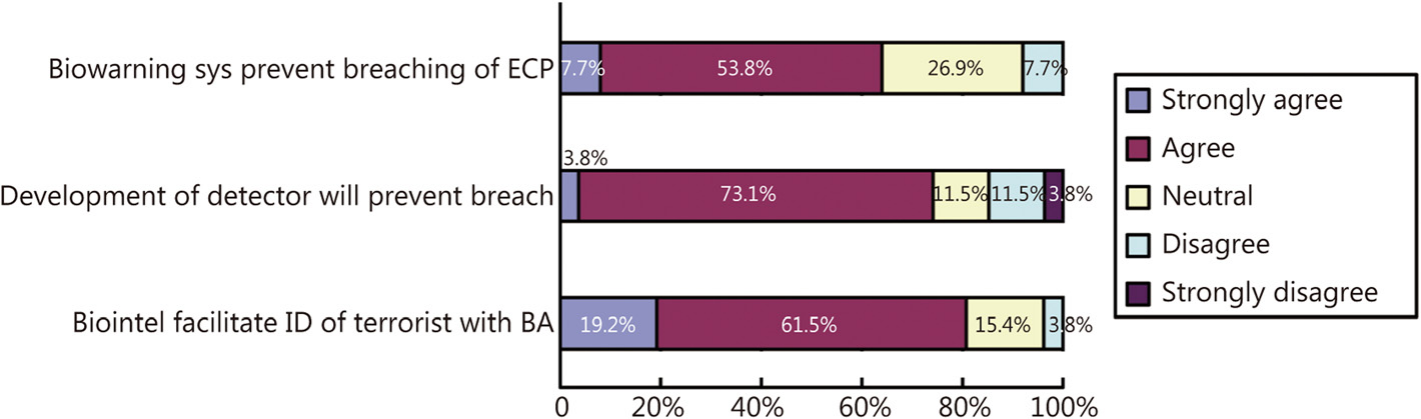
The respondents’ perceptions regarding the effectiveness of biointelligence protocols against a BA breach at an ECP

Slightly more than eighty percent (80.7 %) of the respondents agreed or strongly agreed that biological intelligence would facilitate the effective identification of a terrorist with a bioagent at the ECP. More than seventy-six percent (76.9 %) agreed or strongly agreed that the deployment of biological agent collectors or detectors in the area of operation (AO) would prevent an ECP breach by a terrorist with a bioagent. Finally, 61.5 % agreed or strongly agreed that a biological warfare attack warning system would prevent the breach of an ECP by an HBBA terrorist (Fig. 2).

#### Protective equipment and the breaching of ECPs by an HBBA terrorist (FM 3-11.4)

**Fig. 3:**
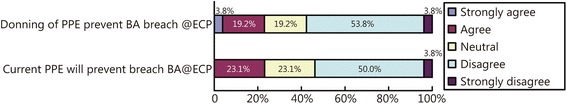
The respondent’s perceptions regarding the effectiveness of personal protective equipment protocols against a BA breach at an ECP

The responses regarding how effective the current CBRN TTP (FM 3-11.4) would be in the detection, deterrence or mitigation of a successful breach of the ECP by an HBBA terrorist were analyzed. Approximately fifty-five percent (53.8 %) of the respondents strongly disagreed or disagreed with the notion that current individual protective equipment would prevent the breach of a combat ECP by a terrorist carrying a bioagent. Fifty-seven percent (57.6 %) strongly disagreed or disagreed with the statement that wearing protective gear would prevent the breach of a combat ECP by a terrorist carrying a bioagent (Fig. 3).

### Respondents suggestion for the modification of current FOB ECP TTPs

In response to the item asking whether there is a need for any modification of current ECP TTPs to enhance security against a human terrorist with a BA at an ECP, 72.0 % of the respondents responded “yes”. Over 70.0 % of those who answered “yes” recommended the need for training and education on bioagents and bioterrorism for every personnel manning the ECP. Over 60.0 % recommended that in addition to training, effective equipment for bioagent detection should be provided for personnel at the ECP.

### Responses regarding BA devices at ECPs

Fifty percent of the respondents strongly disagreed or disagreed with the statement that soldiers at the ECP had devices, such as the explosive trace detector spray kits employed for improvised explosive devices (IEDs) that could effectively detect traces of BAs on a person at the ECP. Similarly, 53.8 % either strongly disagreed or disagreed that every soldier at the ECP is adequately knowledgeable about how to look for BAs or what to look for.

## Discussion

This preresult indicated that 61.5 % of the respondents, the majority of whom were combat veterans, think it is either possible or very possible for a terrorist carrying a biological agent to successfully breach a combat ECP undetected. The fact that the analysis indicated that over 60.0 % of the respondents either agreed or strongly agreed that biological intelligence, the deployment of biological collectors or detectors and biowarning signals would be effective in preventing the breach of a combat ECP by an HBBA terrorist tends to indicate limited knowledge. These policies or practices are more reactive in nature, as they are deployed in response to the detected presence of a released bioagent in line with the U.S. military and government; hence the 2002 National Strategy to Combat Weapons of Mass Destruction (CWMD) [31, 32]. The federal government has deployed biodetectors around certain areas on civil security bases under the “BioWatch” program, as documented in Chapa’s [33] article. Regarding the use of personal protective equipment (PPE) and immunization as currently practiced under the CBRN TTPs, over 50 % and 45 % of respondents, respectively, strongly disagreed or disagreed that these methods would effectively prevent an HBBA terrorist from breaching an ECP. Usually, PPEs are donned after intelligence has indicated a possible threat, generally in war theater and not necessarily at the ECP, while immunization is performed to prevent infections based on the threat of a specific agent that intelligence has identified.

It is striking to note that the respondents claimed that soldiers are not educated regarding searching for BAs at the ECP, nor are they equipped with the necessary devices to detect a BA on a person or a vehicle, such as the equipment employed for IED searches. While the authors acknowledge the limitations of this study, the perceptions of these respondents remain very authentic because they have firsthand combat ECP TTP experiences. During this study, many of the respondents suggested the need for authorities to educate soldiers about bioterrorism and HBBA terrorists and the need for changes in current ECP TTPs to include procedures that involve searching for BAs.

The authors also acknowledges the difficulties that might arise with the development of devices that will effectively detect all BAs; however, the current situation of a complete absence of any BA-deterrent search practices and the use of obsolete biowarfare TTPs, as enumerated in the current FM 3-11 series, is troubling. The successful importation of the Ebola virus into Nigeria and the U.S.A. by individuals who knew they had had contact with people infected with the Ebola hemorrhagic virus remains the most parallel case and the best practical example of a human-borne with bioagent (HBBA), a human mobile bioagent device. Those individuals knew that they were infected, yet lied to the officials at the ECP to enter their intended countries. Those events tend to confirm the feasibility and possibility of an intentional and deliberate transportation of an infectious agent in-borne by humans across borders to cause or spread disease in another country. Mr. Patrick Sawyer was actively incubating a deadly virus in an implanted IED, which is known as a surgically inserted improvised explosive device (SIIED) in military parlance; thus, he was a “human-time-bomb,” an HBBA who left his country undetected by a point of exit/entry (POE) security metal detector search, flew into Nigeria, and passed through metal detectors and other POE security searches that failed to detect or identify the virus he was carrying.

Similarly, Mr. Thomas Eric Duncan cared for a family/people who actively exhibited EVD symptoms and who later died. He lied to officials at the Liberian airport about his prior contact to board an airplane ultimately headed for the U.S., where as many as 100 individual were placed at risk of possible infection [34, 35]. The Liberian president was reportedly unhappy with Mr. Duncan’s behavior and stated his willingness to prosecute the index case when or if Mr. Duncan returns to Liberia [36].

There are no longer any illusions regarding the possibility and feasibility of humans becoming carriers of biological agents, and there are no illusions regarding the actions of Al Qaeda and other terrorist groups, who are actively seeking the means to acquire and transport BAs through the U.S./Mexico border with the intention of causing terror against the United States [37, 38]. Furthermore, the U.S. has numerous military bases on foreign lands, and military personnel can acquire microbial infections during deployment that can be transported to the homeland [22–24]. We cannot wait for another 9-11 attack before accepting that this form of terrorism is feasible, as the enemy has proven to be always to be one step ahead. Now is the time for civil security/national security personnel and leaders to break this circle. It would be a mistake to confuse biowarfare with bioterrorism; the former requires sophisticated equipment that it is not very necessary in the latter, especially when there are willing volunteers seeking martyrdom.

### Significance of study

A successful breach of a U.S. military combat post by a bioterrorist would not only have a devastating effect on morale, it would affect the military’s fighting strength and thus its missions. It would provide an additional route of BA dissemination to the U.S. mainland. The failure to detect a BA during its incubation period would result in a failure to treat infected soldiers (thus making them carriers of the BA), especially those returning home for rest and recuperation (R & R) and those redeploying home. This study was the first to investigate the possible vulnerability of combat FOBs to a breach at the ECP by an HBBA terrorist under current military measures.

### Limitations of the study

This study employed a time-specific, cross-sectional design for the purpose of determining perceptions that are critical to ECP security at a combat post by those who are charged to ensure it. The restrictions placed on the target respondent organizations’ populations, the classification limitations placed on military TTPs, and possible effects on the participants’ responses to the questionnaire are other limitations.

## Conclusion

If obtained from proper sources and communicated to the personnel at the ECP in an effective or timely manner, the effectiveness of certain TTPs outlined in the FM 3-11 - specifically FM 3-11.86, intelligence - might increase. The fact remains that while there are drills on soldiers’ reactions to a biological attack, there is no specific training for the ECP, such as how to search for biological agent in or on a person at the ECP. This explains how the retired air force colonel described by Hylton (29) was able to get a modified anthrax bacillus into the White House and how an Afghani worker was able to enter an FOB and serve food to soldiers despite having an infectious skin problem. At ECPs, therefore, the current FM 3-11 methods would be very ineffective for preventing an HBBA breach.

## Abbreviations

HBBA: Human-borne with bioagent
ECP: Entry control point
BA: Biological agent / bioagent
DoD: Department of defense
BT: Bioterrorism
CBRN: Chemical, biological, radiological, nuclear
FOB: Forward operating base
DIFAC: Dinning facility
LN: Local National
TTP: Tactics, techniques, procedures
PBIED: Person-borne improvised explosive device
VBIED: Vehicle-borne improvised explosive device
AT: Anti-terrorism
OEF: Operation Enduring Freedom
FM: Field manual
SIIED: Surgically inserted improvised explosive device
